# An *In-Situ* Corrosion Small Punch Test for Developing Stress Corrosion Cracking in Stainless Steel

**DOI:** 10.1007/s11340-025-01177-y

**Published:** 2025-03-27

**Authors:** K. Yuan, M. Mokhtarishirazabad, S. Mckendrey, R. Clark, M. Peel, M. Mostafavi

**Affiliations:** 1 Univeristy of Bristol, University Walk, BS8 1TR Bristol, United Kingdom; 2 National Nuclear Laboratory, 102B Stonehouse Park, Sperry Way, GL10 3UT Stonehouse, United Kingdom; 3 Monash University, 17 College Walk, Melbourne, Victoria 3168 Australia

**Keywords:** Stress corrosion cracking (SCC), Small punch test (SPT), Stainless steel, Materials characterisation, Mechanical properties

## Abstract

**Background:**

Spent AGR (advanced gas-cooled reactor) fuel cladding may suffer from stress corrosion cracking (SCC) during the interim storage period in cooling ponds and compromise the structural integrity of fuel storage.

**Objective:**

To better understand the effect of SCC, a new small punch test (SPT) setup was developed in this study that can use a small volume of sample to limit the safety concerns about irradiated materials.

**Methods:**

The SPT setup accelerated SCC in a surrogate material 304 stainless steel by introducing a circulation of a heated corrosive solution. Preliminary tests were performed to find the loading and environmental conditions that can develop SCC in the surrogate material. A finite element model was used to estimate the mechanical behaviour of the material during the test.

**Results:**

Several samples were tested under different conditions, and SCC and other forms of corrosion behaviours were observed on the samples. The effects of different corrosive environments were obtained by further characterisation including scanning electron microscopy (SEM) and optical profilometry.

**Conclusions:**

The experiment demonstrated the new setup can develop SCC from a small volume of sample in a short period of time. Several improvements are listed including extra procedures to enable the experiments on the irradiated fuel materials.

## Introduction

Stress corrosion cracking (SCC) commonly occurs in austenitic alloys in a corrosive environment, which could cause premature failure of structural materials in different scenarios in the nuclear industry. This phenomenon is caused by the combination of a susceptible material, a mechanical driving force and a corrosive environment [1, 2]. The initiation of SCC is often found on the surface defects of the materials such as pits, inclusions, coating defects and pre-existing cracks [3–5]. SCC can often be found in the components of the water circuits in light water reactors such as PWRs (pressurised water reactors) and BWRs (boiling water reactors) [6, 7]. When spent AGR (advanced gas-cooled reactor) fuel is stored in cooling ponds, IGSCC (intergranular stress corrosion cracking) may occur on the fuel cladding and lead to failure of the whole fuel pin [8, 9]. The AGR fuel pin employs custom-made austenitic stainless steel 20/25/Nb (chemical composition in Table 1), where niobium is introduced to the alloy to reduce the concentration of carbon in the matrix by forming niobium carbide [10]. The lower matrix carbon concentration can mitigate the effects of thermal sensitisation and preserve the corrosion resistance by reducing the formation of chromium carbide and preserving the chromium concentration near the grain boundaries [11, 12]. However, during the operation, neutron irradiation can cause RIS (radiation-induced segregation) by redistributing the atoms near grain boundaries [13, 14], which causes the depletion of chromium (to around 12%) near the affected grain boundaries [15] like thermal sensitisation and reduces the corrosion resistance of the cladding material. When the compromised fuel cladding is stored in the cooling ponds, the combination of the residual stresses in the material and the chloride in the ponds may cause the initiation of stress corrosion cracking [8, 16], which can potentially lead to the failure of the fuel pin. A mechanical testing method is required to test and monitor the development of radiation-induced SCC to better understand the phenomenon while the cladding is stored in the ponds.

**Table 1:** Chemical compositions (wt%) of the surrogate 304 stainless steel and the fuel cladding material 20/25/Nb [27, 28]

Material	Cr	Ni	P	Si	Mn	Nb	S	C	Fe
304	18	10	0.045	0.5	1	-	0.015	0.07	Bal.
20/25/Nb	20	25	0.045	0.75	2	0.055	0.03	0.05	Bal.

**Table 2:** Grain sizes, mechanical properties and sensitisation properties of irradiated 20/25/Nb ($\sim$ 20 GWd/te at 420 $^{\circ }$C) and thermally-sensitised 304 stainless steel (data from [8, 29, 30])

Materials	Grain size	Yield strength	Young’s modules	Lowest Cr content	Cr depletion width
20/25/Nb	16 ± 3 $\upmu$m	220 MPa	198 GPa	12%	$\sim$ 40 nm
304	23 ± 8 $\upmu$m	253 MPa	211 GPa	10%	$\sim$ 60 nm

Various mechanical testing methods and techniques have been applied with corresponding environmental conditions to develop and study SCC. Traditionally, tensile and bending tests are commonly used to study SCC. A constant load or strain can be applied to an intact or pre-cracked tensile sample to accelerate the process [17–19]. Three-point and four-point bending tests are often performed as the deflection and applied stresses can be easily adjusted [20, 21]. However, these testing methods require a larger sample size, and these samples cannot be made from the fuel cladding due to its cylindrical geometry and small diameter of 16 mm. Micro-mechanical testing methods have also been performed to study SCC by loading a FIB (focused ion beam)-milled micro-cantilever containing a grain boundary [22], these methods however may not represent the mechanical behaviours of the bulk material [2]. The small punch test (SPT) is an ideal method for this scenario, as only a small disc sample is required to perform the test, which can be taken from the end cap of the cladding. This method is commonly used in the nuclear industry as it can obtain different mechanical properties including yield strength, tensile strength, fracture toughness, fatigue and creep behaviours in different conditions [23, 24]. Isselin et al. [25] performed SPT to test stress corrosion cracking of 316L stainless steel in a simulated BWR environment (at 288 $^{\circ }$C and 9 MPa), and SCC was developed over a few days by applying a cyclic load, which might have introduced other forms of cracking. A new SPT setup was developed by Yu et al. [26] where a circulation of synthetic seawater was pumped to the centre of a square sample, and a load with a slow strain rate was applied until fracture. This test can only show the relative susceptibility of SCC at different loads instead of developing SCC in real time.

This research presents a small punch test setup that can initiate SCC with the circulation of a corrosive solution. Due to the accessibility of the irradiated fuel cladding, a surrogate material thermally-sensitised 304 stainless steel was used to demonstrate the new setup because of its similar chemical compositions (shown in Table 1) and mechanical properties (shown in Table 2). Preliminary tests were initially carried out to find the loading and environmental conditions for developing SCC. The corrosion small punch tests were performed under different conditions, and SCC was successfully developed. The sample was compared with a finite element model to find the relationship between stress and SCC. Finally, the samples were further characterised by scanning electron microscopy (SEM) and optical profilometry to observe the corroded regions.

## Experimental Methods

### Materials and Sample Preparation

**Fig. 1:**
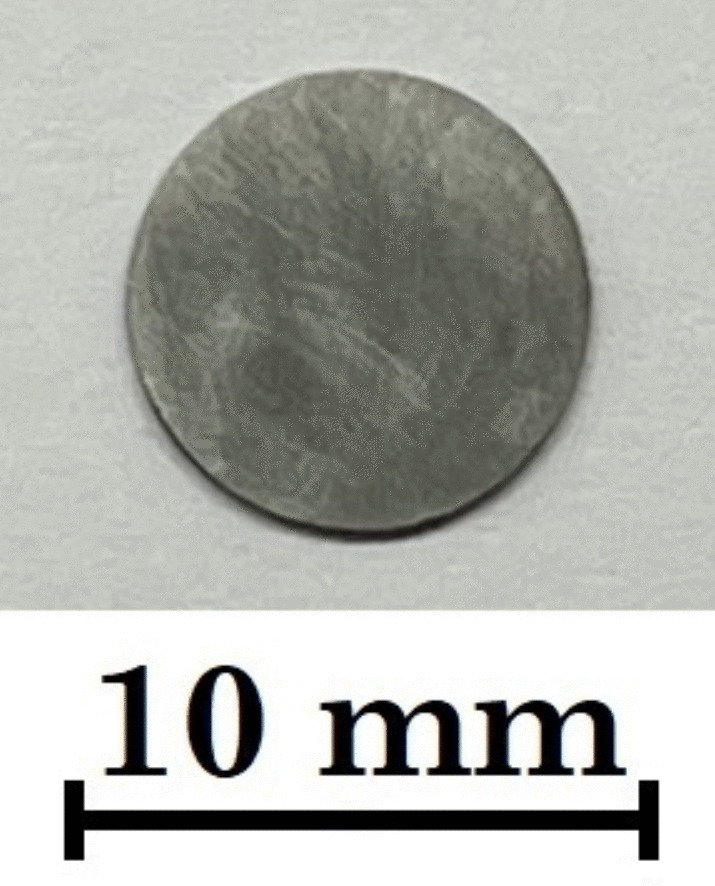
Prepared disc sample for the corrosion small punch test

As-received 304 stainless steel sheets (chemical composition shown in Table 1) were initially thermally sensitised by ageing at 600 $^{\circ }$C for 50 hours in air [8]. A heat-treated material was electro-etched at 6 V in 10 % wt oxalic acid for 1 minute, and more than 85% of the grain boundaries showed chromium carbide precipitation by optical image analyses marking significantly reduced corrosion resistance after the heat treatment. Several SPT disc samples were cut from the heat-treated sheets by EDM (electrical discharge machining). According to the CEN (European Committee for Standardization) standard [31], each sample had a diameter of 8.05 mm and a thickness of 0.5 ± 0.005 mm. The sample surface (shown in Fig. 1) was initially ground to 1000 grit and then scratched with 600-grit sandpaper randomly to accelerate the corrosion process of the samples.

### Small Punch Test Setup

**Fig. 2:**
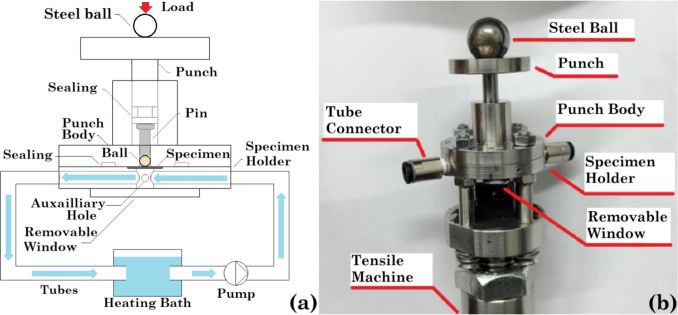
(a) Schematic of the novel small punch test setup with the circulation of the corrosive solution (highlighted in blue) and (b) manufactured SPT rig

Figure 2 (a) shows the design of the new SPT setup containing three parts: the SPT rig, a heating bath and a pump, and the manufactured rig is shown in Fig. 2 (b). The SPT rig was designed based on the CEN standard [31], and it consisted of three main components: the specimen holder, the punch body and the punch, which were made of corrosion-resistant 316L stainless steel. The specimen holder had a recess with a diameter of 8 mm and a depth of 0.35 mm for the sample, and the centre bore had a diameter of 4 mm with a 0.2 mm chamfered edge at 45$^{\circ }$. The sample was placed in the recessed area between the specimen holder and the punch body and secured by eight bolts and nuts. The SPT setup was loaded onto a tensile machine with a maximum load of 10 kN, and two load cells with a range of 10 kN and 1 kN were used accordingly. The ambient temperature ranged from 18 $^{\circ }$C to 22 $^{\circ }$C, and the relative humidity ranged from 40% to 70%. A steel ball was placed between the compression head of the tensile machine and the punch of the rig to reduce the effects of misalignment. The load was applied from the punch through a stainless steel pin to the ceramic ball with a diameter of 2.5 mm. The corrosive solution with a volume of 300 ml was heated in a beaker in the heating bath and pumped through a channel in the specimen holder by a peristaltic pump at a flow rate of 100 ml/min. The temperature of the solution was monitored by a corrosion-resistant K-type thermocouple with a stainless steel sheath through the auxiliary hole on the side of the specimen holder. The rig was sealed with O-rings on the punch and between the specimen holder and the punch body. A removable window was placed and sealed below the specimen for quick inspection and cleaning. All the tubes and connectors were made from corrosion-resistant materials, and a filter was placed on the outlet of the tube in the heating bath.

**Fig. 3:**
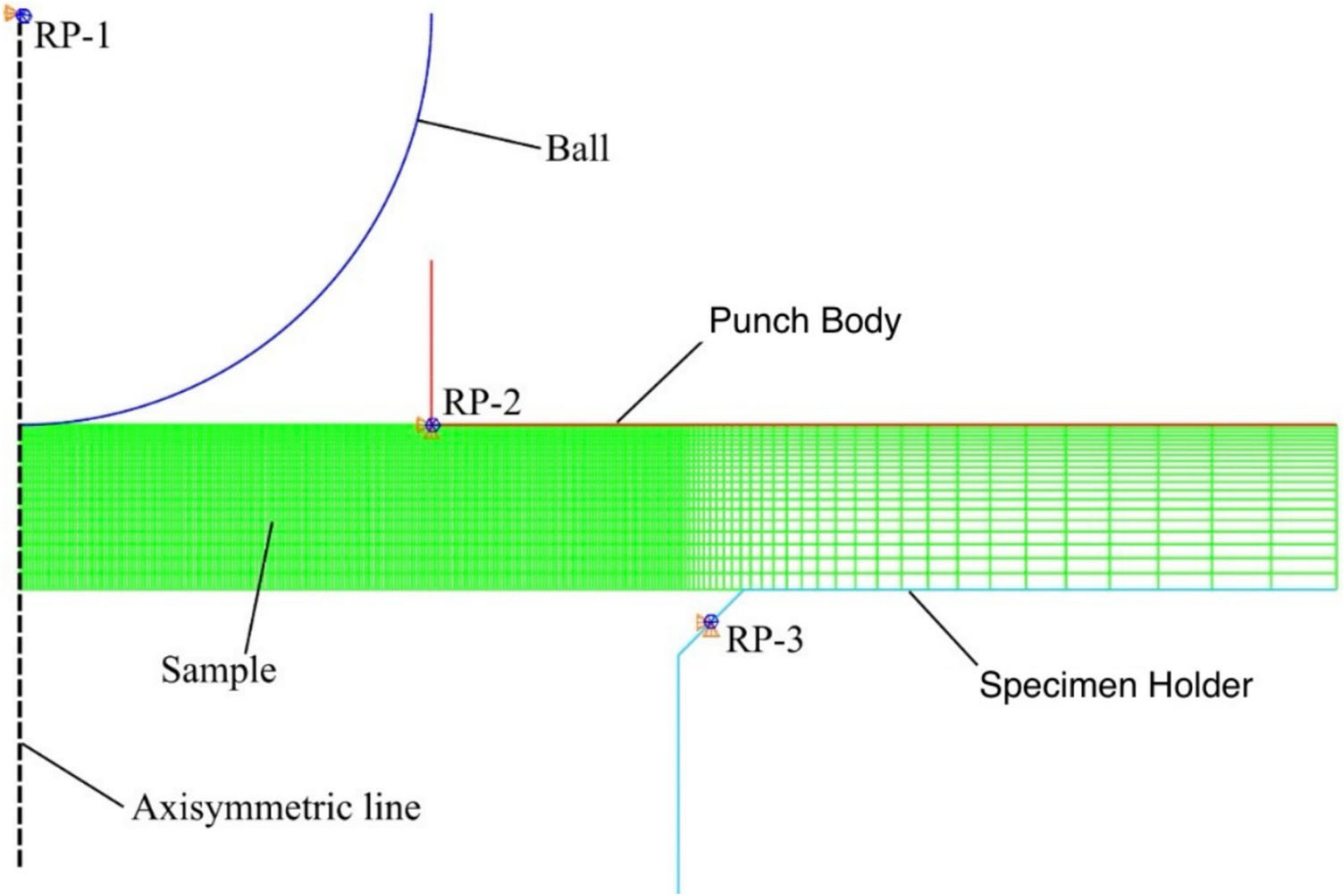
2D axisymmetric finite element model with the sample and three rigid parts with corresponding reference points: ball (RP-1), punch body (RP-2) and specimen holder (RP-3). Boundary conditions are shown as orange cones for linear displacements, and blue cones for rotational displacements

### Preliminary Test

As stress corrosion cracking occurs due to the combination of a susceptible material, a mechanical driving force and a corrosive environment, it was vital to find the loading and the environmental conditions in order to initiate SCC in a short period of time. A preliminary mechanical test was performed by loading a sample to failure without the corrosive environment to find the loading conditions for the corrosion tests. The sample was loaded under displacement control with a displacement rate of 0.3 mm/min until the failure of the sample, and the load and displacement measurements of the tensile machine were recorded. To find an environment that can accelerate the corrosion process, different corrosive conditions were tested and compared. Several small tensile samples were cut and prepared the same way as the SPT disc samples. The samples were loaded in self-contained tensile rigs at the same torque by a torque wrench and placed in a beaker with a solution in the heating bath. They were exposed to two corrosive environments for 7 hours: (a) 1000 ppm sodium thiosulphate solution at 55 $^{\circ }$C and (b) 1000 ppm sodium thiosulphate solution with 1440 ppm hydrochloric acid at 70 $^{\circ }$C [8] [32]. The samples were inspected with an optical microscope at the end of exposures.

### Finite Element Model

As the small punch test is not a uni-axial testing method, the stresses on the sample cannot be easily calculated based on the applied force and deformation. In order to find the stresses that contributed to the initiation of SCC in the sample under different loading conditions, a finite element model [33–35] was used in Abaqus CAE [36]. The sample and experimental setup were modelled as 2D axisymmetric parts (shown in Fig. 3). The sample with a dimension of 4 $\times$ 0.2 mm was meshed using 4620 CAX4R deformable four-node bilinear axisymmetric quadrilateral elements, with element width in the region of interest of 10 $\upmu$m, and height of 10 $\upmu$m at the top surface of the sample increasing to 49.61 $\upmu$m at the bottom surface. The ball, punch body and specimen holder were modelled as RAX2 rigid two-node linear axisymmetric elements, so that the meshes aligned with those of the sample to ensure good contact convergence. Each rigid body part was tied to a reference point, and the boundary conditions were then applied to the reference point. The punch body and specimen holder had a zero displacement boundary condition applied to both x and y directions and rotation about the z-axis. The ball had a zero displacement boundary condition applied only in the y-direction and rotation about the z-axis.

The surface to surface discretization was applied to the contact between the sample surface and the ball, and the Coulomb friction coefficient was assumed to be $\upmu$=0.075 due to the smooth surface finish of the ball and the sample [33, 37]. All other surface interactions were assumed to be frictionless. The ball was in contact with the sample at the beginning of the model. A point load was applied to the reference point (RP-1) associated with the ball, over four steps, using a dynamic-explicit time integration method. The load increased at each step at 50 N, 100 N, 150 N and 200 N. The material used for the ball had an elastic modulus of *E* = 211 GPa, *v*=0.3, and initial yield strength of $\sigma _y$=253.4 MPa.

### Corrosion Small Punch Test

Four samples were tested by corrosion small punch tests with different combinations of sample preparation methods and testing conditions (Table 3). The tensile machine was controlled under load control mode, and a constant load was applied to the sample. Based on the results of corrosion preliminary tests, a corrosive solution with 1000 ppm sodium thiosulphate and 100 ppm hydrochloric acid was heated to different temperatures and circulated in the loop of the SPT setup. The temperature of the sample was recorded and kept at a constant value. The samples were under continuous exposure and inspected periodically by an optical microscope, and the tests were terminated when corrosion or cracking was observed on the sample.

**Table 3:** Corrosion small punch test conditions

Sample	1	2	3	4
Load (N)	200	200	200	200
Sample temperature ($^{\circ }$C)	60 ±3	55 ±3	45 ±2	45 ±2
Duration (hours)	45	94	66	209

**Fig. 4:**
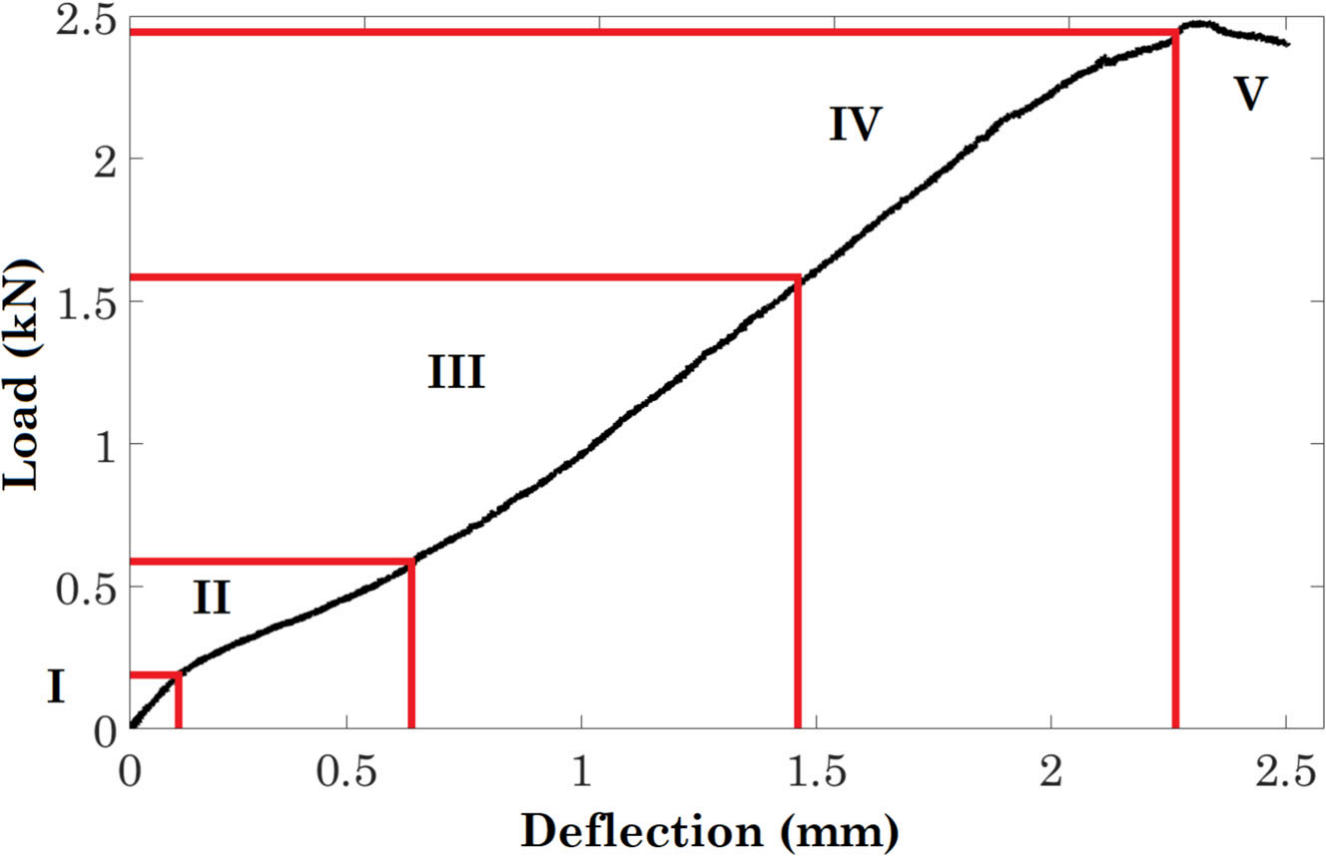
Load and deflection curve of the preliminary small punch test without the corrosive environment. Five regions on the curve: I elastic bending, II plastic bending, III membrane stretching, IV plastic instability, and V unstable yielding and failure (the inflection point between stage III and VI is an estimate)

### Sample Characterisation

Once corrosion or cracking was detected using an optical microscope, the sample was cleaned and further inspected by SEM under secondary electron and backscattered electron modes. Sample 2 was cut across the corroded regions and observed. Sample 1 and sample 3 were observed with optical profilometry by capturing images of the sample at different focal lengths, and an Alicona Infinite Focus system was used with two settings. The full field of both samples was scanned at a lower $5\times$ magnification, and 4 sets of images were taken to cover the sample. The images had a vertical resolution of 429 nm and a lateral resolution of 7.82 $\upmu$m. The cracked region of sample 3 was mapped at a higher $20\times$ magnification with 1 image to cover the area. A higher vertical resolution of 51 nm and a lateral resolution of 2.93 $\upmu$m was applied. A 3D volume of the surface was reconstructed, and line scans were taken across the cracks and pits to measure the depths of them.

**Fig. 5:**
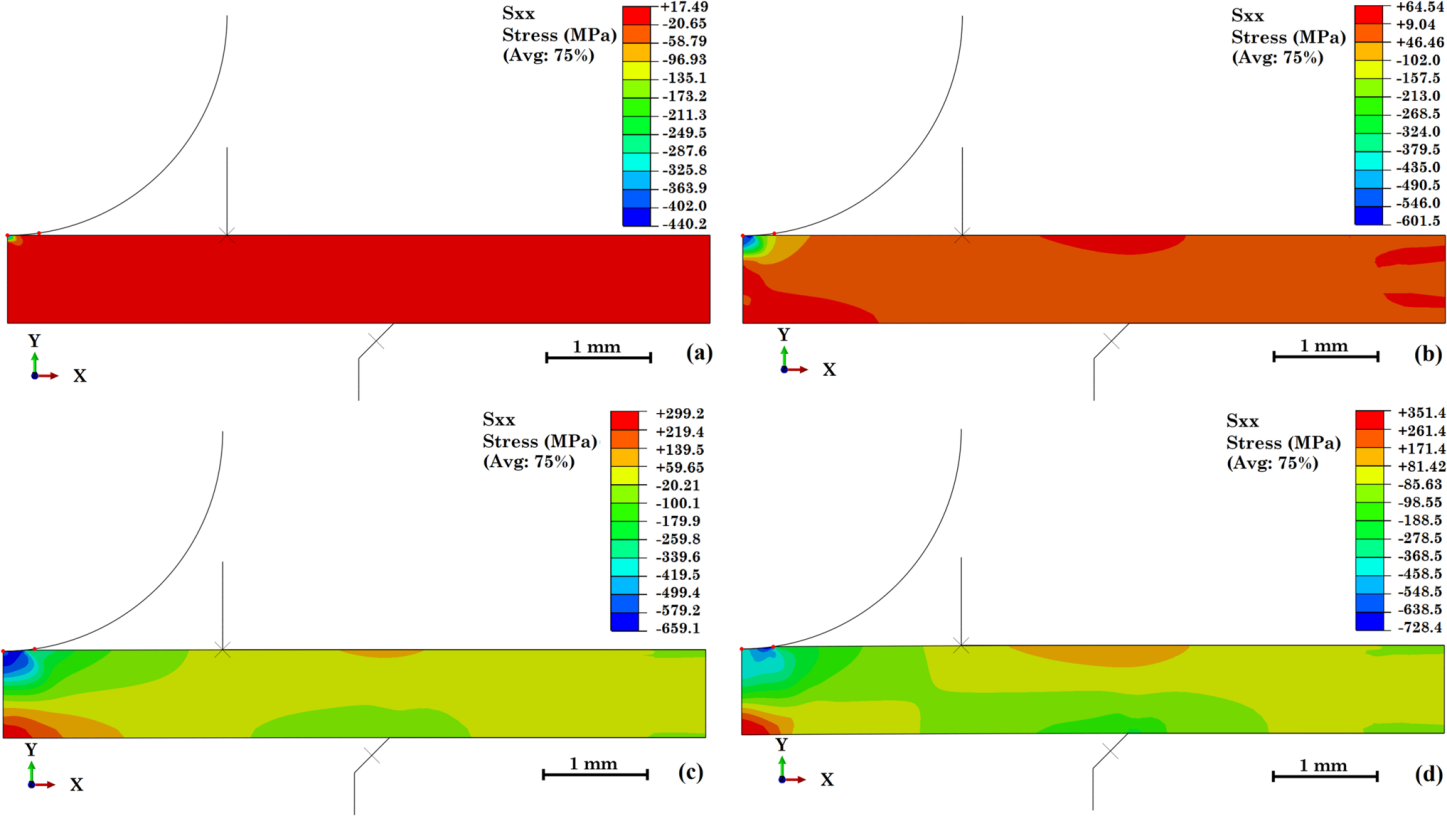
$S_{xx}$ direction stress maps of the finite element model under (a) 20 N, (b) 50 N, (c) 100 N, and (d) 200 N

**Fig. 6:**
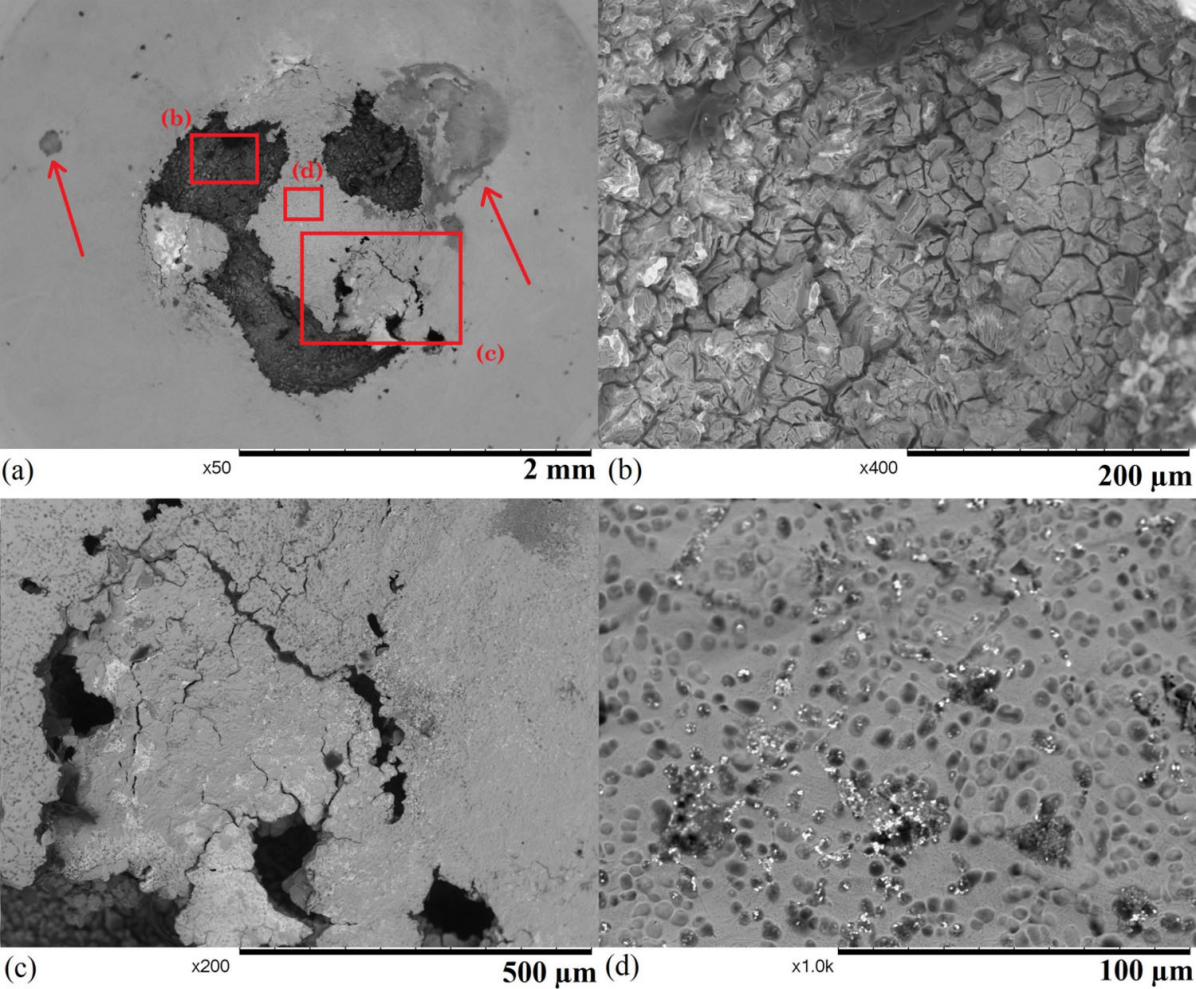
SEM images of sample 1 after 45 hours of exposure: (a) an overview of the sample with crevice corrosion sites highlighted, (b) a void that suffers from mixed forms of corrosion, (c) a region that dominantly suffers from stress corrosion cracking, and (d) the surface with shallow pits

**Fig. 7:**
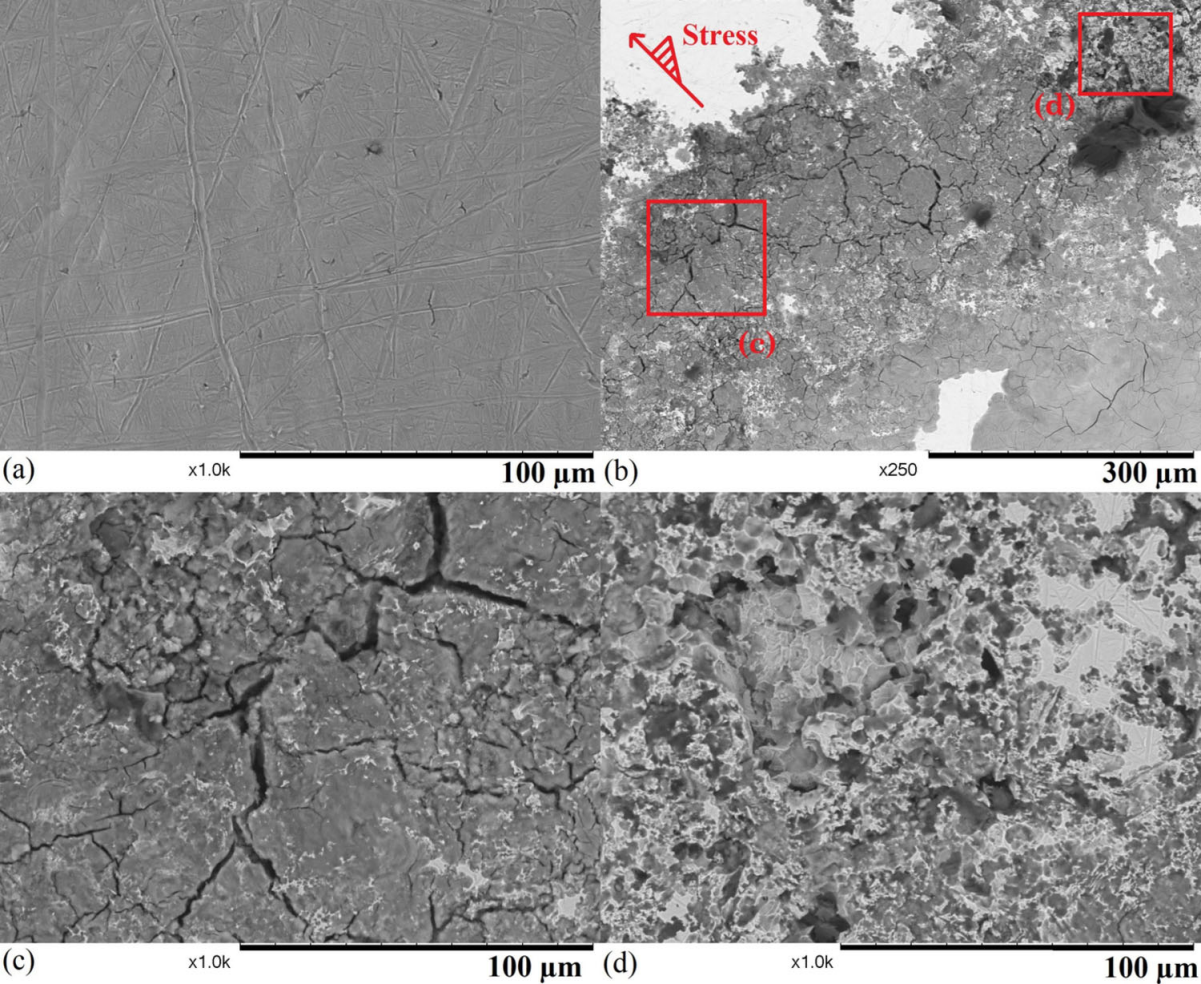
SEM images of sample 2 after 94 hours of exposure: (a) an uncorroded region, (b) a region that suffers from mixed forms of corrosion with stress gradient shown by an arrow, (c) a region that suffers from stress corrosion cracking, and (d) a region that suffers from pitting corrosion

## Results and Analyses

### Preliminary Test

Figure 4 shows the load and deflection curve of a trial small punch test without the corrosive environment. The deflection of the centre of the sample is assumed to be the same as the displacement of the compression head of the tensile machine. The graph shows five stages of the small punch test: I: elastic bending, II: plastic bending, III: membrane stretching, IV: plastic instability and V: unstable yielding and failure. During stage I and stage II, the transition from elastic deformation to plastic deformation of the sample occurs, and a yielding load of 210 N can be identified at the transition point. At these stages, the contact area is increased with deflection, but the thinning effect does not occur [38]. During stage III and stage IV, the reduction of the sample thickness starts due to the thinning effect causing the initiation and coalescence, which leads to the fracture of the sample. Note that, unlike other inflection points, the transition between stage III and stage IV does not show a significant change in the gradients, and the point only represents an approximate transition between the two stages. This was possibly caused by the reduction of imperfections from the heat treatment. Because the AGR fuel cladding only suffers from elastic stresses such as residual and thermal stresses in storage, the deformation of the corrosion SPT samples should be kept in the elastic region. A load of 200 N was therefore chosen for the corrosion small punch tests.

### Finite Element Model

Figure 5 shows the stress distribution of the small punch test under different loads. Only normal stress in the $S_{xx}$ direction is analysed, as it is the dominant stress that opens the surface SCC. At lower loads (20 N and 50 N), only the region that contacts the ball suffers from compression in the $S_{xx}$ direction, and the rest of the sample experiences relatively uniform low tensile stresses. At 20 N, the whole sample suffers from a tensile stress of 17 MPa, and at 50 N, the centre region of the sample experiences a higher tensile stress of 65 MPa, which are both below the yield strength of the heat-treated 304 stainless steel. At a higher load, the centre of the lower surface of the sample experiences stresses beyond the yield strength of the material: 300 MPa under 100 N and 350 MPa under 200 N. The compressive stress at the contact region barely increases, as the sample plastic deformation starts due to yielding. The finite element models show that the SPT sample experiences a complex stress distribution during tests. Even a load lower than the yield point of the test can cause yielding at a local level.

**Fig. 8:**
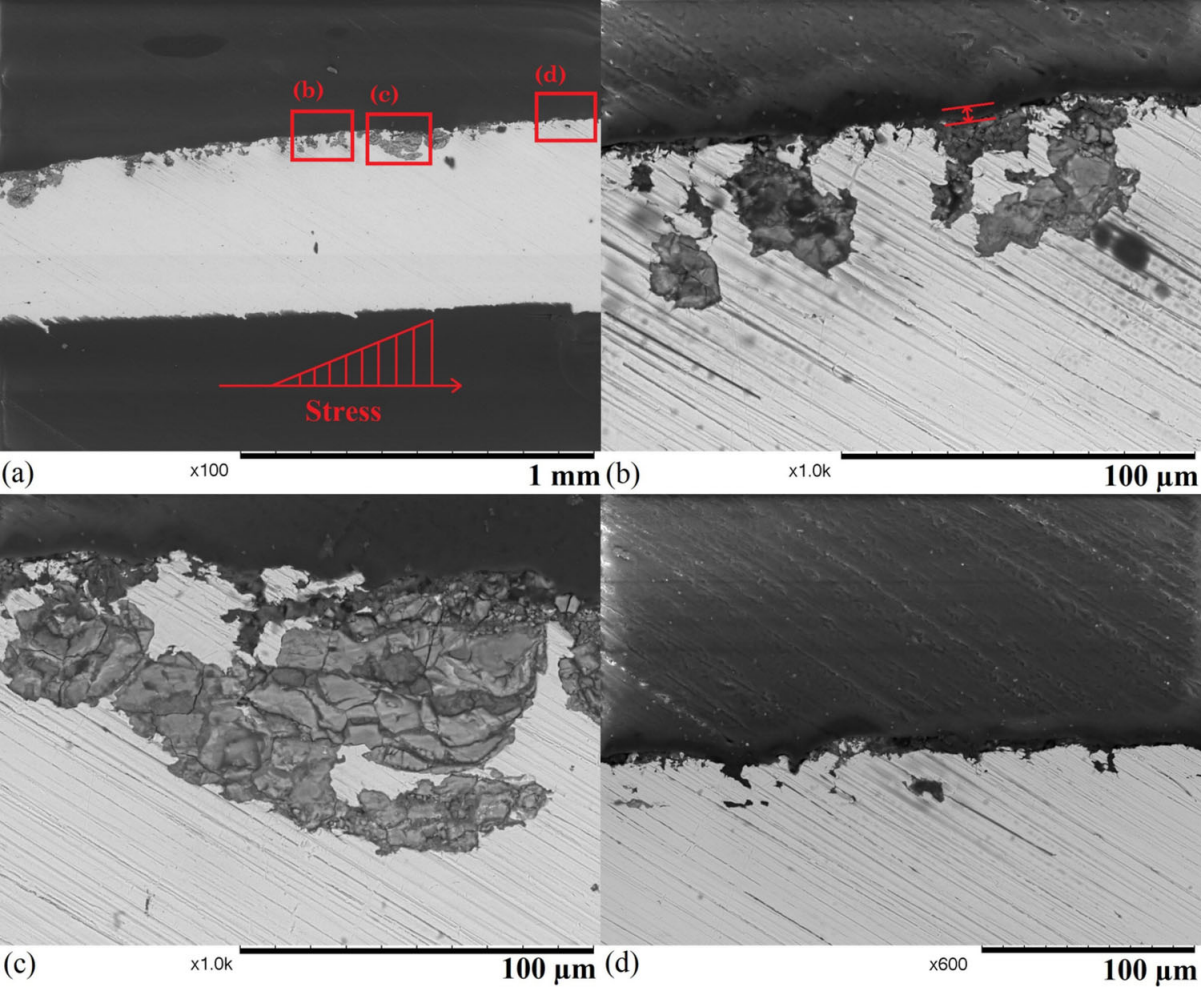
Sideview SEM images of sample 2 after 94 hours of exposure: (a) an overview of the corroded region, (b) a region with several discontinuous corroded areas with the depth of surface general corrosion shown, (c) a large region that suffers from sub-surface corrosion, and (d) a region that suffers from pitting corrosion

### SEM Observation

Figure 6 shows the surface of sample 1 after 45 hours of exposure; various forms of corrosion are found on the sample. These corrosion features only appear in the centre of the specimen with a diameter of 2 mm corresponding to the region that suffers from the highest stress in the FE model (shown in Fig. 5 d), which suggests that the stress was a vital driving factor of corrosion in the region. Several large voids are formed on the exposed area (shown in Fig. 6 a), and the diameter of each void is around 1 mm. Two voids on the left of the sample coalesce forming a larger void. Inside a void (shown in Fig. 6 b), individual grains are exposed due to a mixture of intergranular corrosion (IGC) and intergranular stress corrosion cracking (IGSCC). When IGC and IGSCC develop and propagate inside the material, a volume of material is detached when it is encapsulated by the IGC/IGSCC, and the same process repeats to form a deep void. Figure 6 (c) shows the growing process of a void. In this region, the material below the surface is corroded and detached from the sample. The surface of the area suffers from a mixture of IGC and IGSCC with only a small volume of material attached to the remaining part of the sample. Once the bridging material is further attacked, the region will be separated from the sample. Some shallow and wide pits are distributed uniformly across the surface of the whole sample (shown in Fig. 6 d), and they are very likely the initiation points of IGC and IGSCC. There are several areas between the sample and the holder that suffer from crevice corrosion (highlighted in Fig. 6 a), but the depth of these corrosion sites is relatively shallow. Overall, sample 1 was heavily corroded due to the combination of the chemical concentration and high temperature.

**Fig. 9:**
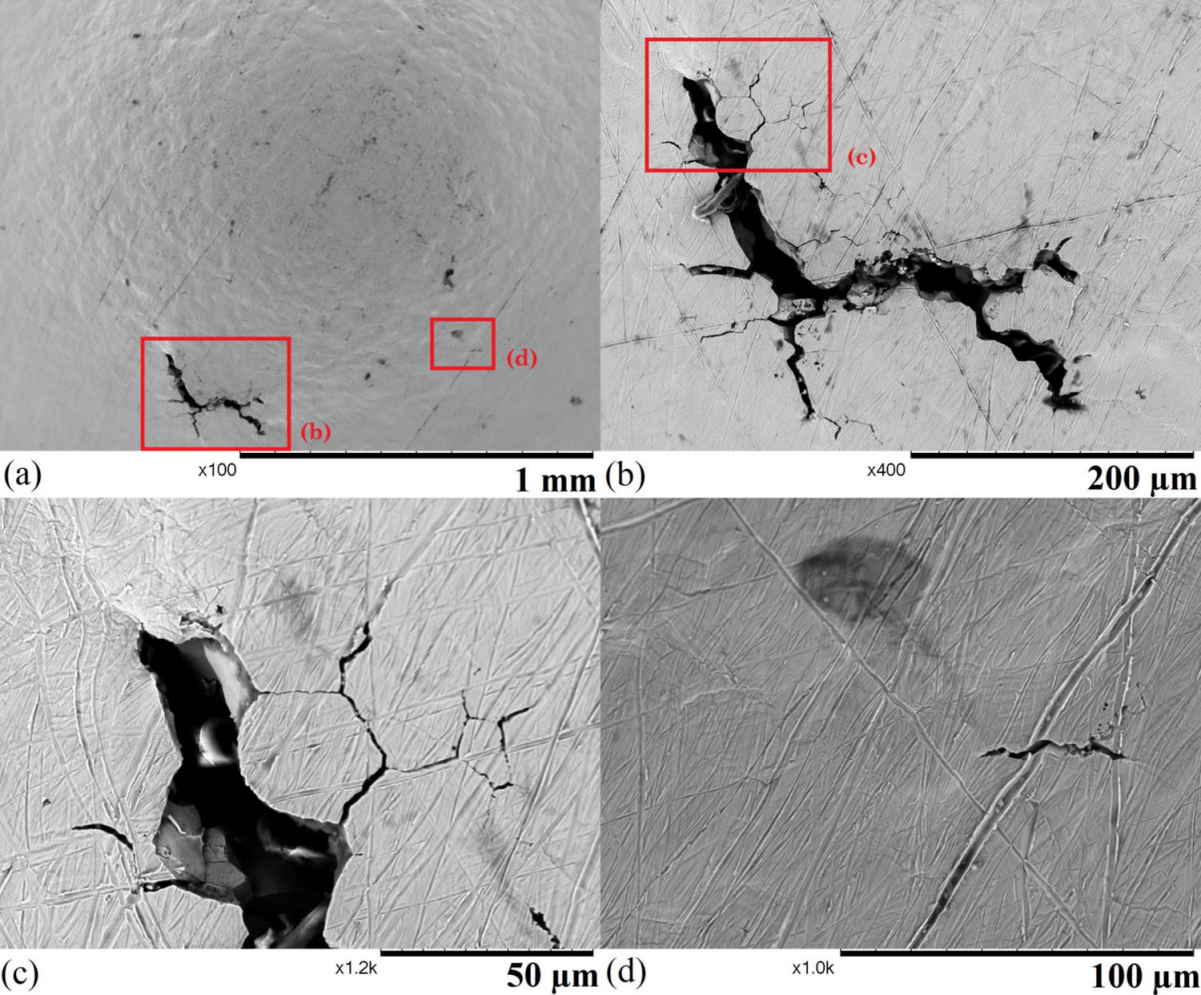
SEM images of sample 3 after 66 hours of exposure: (a) the centre region of the SPT sample, (b) a major cluster of cracks, (c) a tip of the major crack, and (d) the secondary crack site on the sample

Fewer corroded areas can be found on the surface sample 2 (shown in Fig. 7), and most of the sample remains uncorroded with some imperfections brought by the sample preparation (shown in Fig. 7 a). A region showing mixed forms of corrosion with a width of $\sim$ 500 $\upmu$m that is located at the edge of the deformed dome with a diameter of 2 mm due to the high-stress level, which is similar to the centre region of sample 1 (Fig. 1 6 a). The majority of the region suffers from general corrosion, where the sample experiences uniform attack [39], and some stress corrosion cracks can be found in the region. As the centre of the sample is located in the top left corner of the image, there is a stress gradient on the sample surface (marked by the arrow), and the region that suffers from higher stress has more and wider cracks than the region under lower stress. In the region that suffers from higher stress (shown in Fig. 7 (c)), both wide and narrow cracks can be found, and they all show intergranular propagation behaviours. For intergranular stress corrosion cracks, crack widening may occur not only because of higher stress but also heavily attacked grain boundaries. Some grain boundaries experience more severe thermal sensitisation, which can more easily allow the propagation of cracks when SCC occurs. Pitting corrosion is concentrated in the region that suffers from higher stress (shown in Fig. 7 (d)). The stress level however is generally not considered to have a significant influence on the presence of pitting corrosion [40]. The sample deformation may cause a local high air concentration from the circulation system, as the environment is the dominant factor of pitting corrosion. The side profiles of the corroded areas are revealed by cutting the sample in Fig. 8. The overview of the corroded region (Fig. 8 (a)) shows the sample suffers from mixed forms of discontinuous subsurface corrosion, and all the features are relatively shallow with a depth of less than 100 $\upmu$m. The subsurface behaviours of these corrosion features do not show a strong correlation with the stress gradient, as two large corroded regions reside in a low and a high-stress area. Figure 8 (b) shows the general surface corrosion is relatively shallow with a height of less than 5 $\upmu$m. IGC and IGSCC are developed from the corroded sample surface, and most of them are narrow and short (< 5 $\upmu$m). The inside of the material is heavily attacked around several opened cracks or grain boundaries. When many open cracks are present in a small area, the whole region can be heavily corroded below the surface (shown in Fig. 8 (c)). Pitting corrosion features can be found in the regions with less surface general corrosion (shown in Fig. 8 (d)), and they are all relatively shallow with a depth of no more than 20 $\upmu$m. Overall, sample 2 suffers from less severe corrosion than sample 1, but other forms of corrosion still occur.

**Fig. 10:**
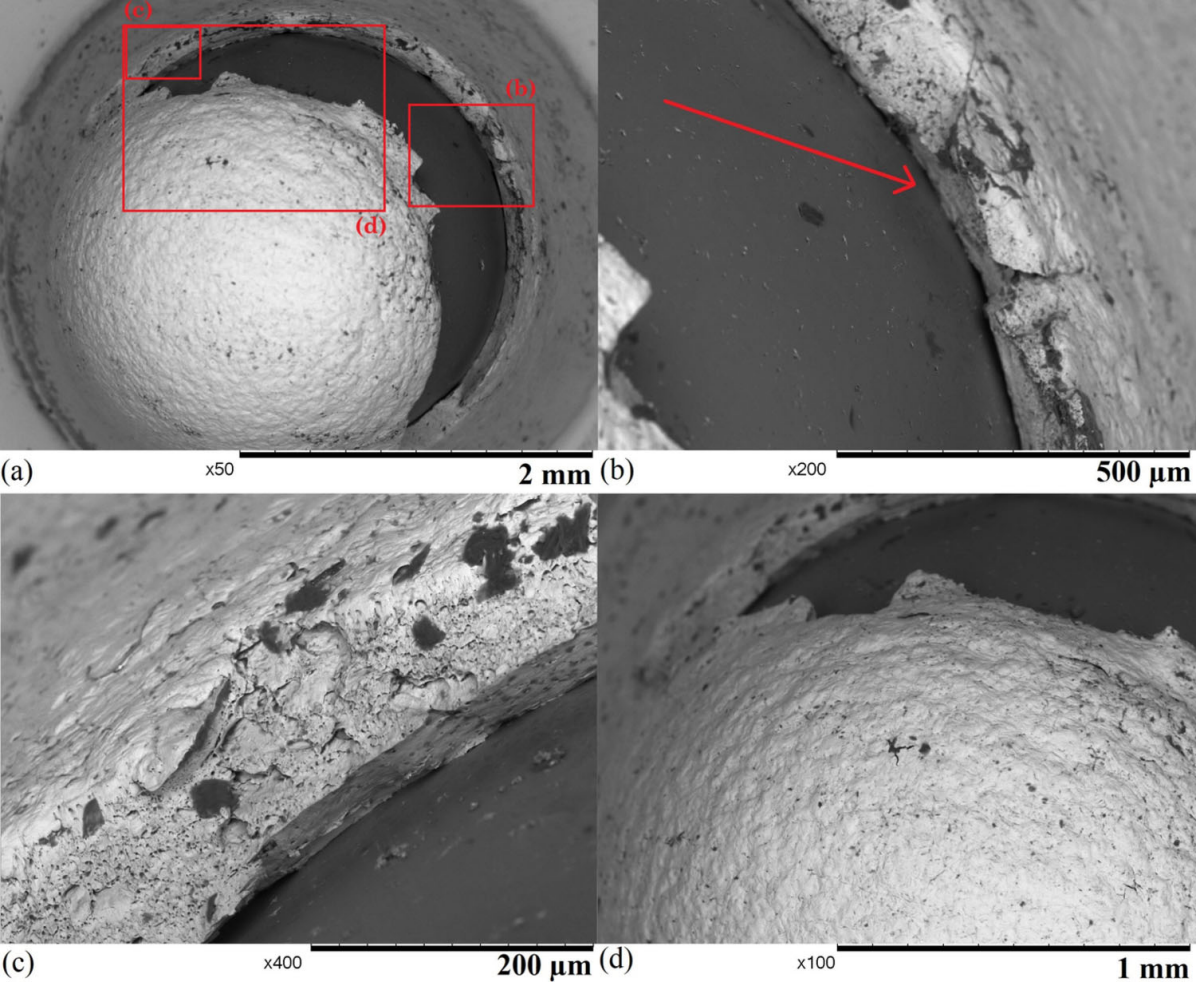
SEM images of sample 4 after 209 hours of exposure: (a) an overview of the failed sample, (b) possible initial point of fracture (highlighted), (c) an area with intergranular corrosion on the fracture surface, and (d) a smaller crack on the sample surface

Only IGC and IGSCC are found on the surface of sample 3 after 66 hours of exposure (shown in Fig. 9 (a)). Unlike the corroded centre regions of sample 1 and sample 2, the corrosion features of sample 3 are all located at the edge of the deformed dome with a diameter of (2 mm), and the centre remains relatively intact. This indicates that a large stress difference in the transition region (shown in Fig. 5 (d)) is the dominant factor causing the initiation of IGC and IGSCC. The major IGSCC site consists of several smaller cracks (shown in Fig. 9 (b)). The overall length of the cracks is 350 ± 30 $\upmu$m, and widths of them range from 5 ± 1 $\upmu$m to 50 ± 3 $\upmu$m. Figure 9 (c) shows the propagation of an IGSCC: intergranular corrosion occurs on corrosion-susceptible grain boundaries due to thermal sensitisation, and the crack grows by propagating along these weakened grain boundaries. A crack preferably propagates in a path with lower energy, and its path is also determined by the loading conditions. In the region shown in Fig. 9 (c), the crack does not propagate through some corroded grain boundaries, which is possibly due to the bridging effect caused by the intact grain boundary below the surface. This also suggests the subsurface propagation of SCC may vary significantly from the surface propagation due to the variation of corrosion-susceptible grain boundaries. Several shorter cracks appear on the regions that suffer from similar levels of stress (shown in Fig. 9 (d)). These shorter cracks did not become the dominant crack because the major crack was initiated earlier. Overall, the combination of the chemical concentration and a lower temperature can accelerate IGC and IGSCC without promoting other forms of corrosion such as pitting and general corrosion.

A repeat test was performed on sample 4 with the same environmental and loading conditions as sample 3 for 209 hours, and the sample came to a complete failure. Figure 10 (a) shows that the sample has a typical SPT failure of a ductile material, where the widely-opened fracture surfaces span hemispherically across the deformed region. The crack had possibly initiated in a region shown in Fig. 10 (b), which is located in the centre of the opening. The irregular shape of the fracture surface (highlighted Fig. 10 (b)) is likely a large stress corrosion crack that does not propagate perpendicular to the sample surface. The sample fails when the initial SCC grows to a critical point, as other parts of the sample show a clean and ductile fracture surface. Intergranular corrosion is observed on a region of the fracture surface (shown in Fig. 10 (c)), and the area is relatively small with a dimension of 160 $\upmu$m $\times$ 80 $\upmu$m. Several shorter cracks can be found on the deformed surface of the sample (shown in Fig. 10 (d)), and similar to the smaller cracks of sample 3, these cracks did not become the dominant crack causing the failure. Sample 4 shows the repeatability of the new method with the given combination of loading and environmental conditions.

**Fig. 11:**
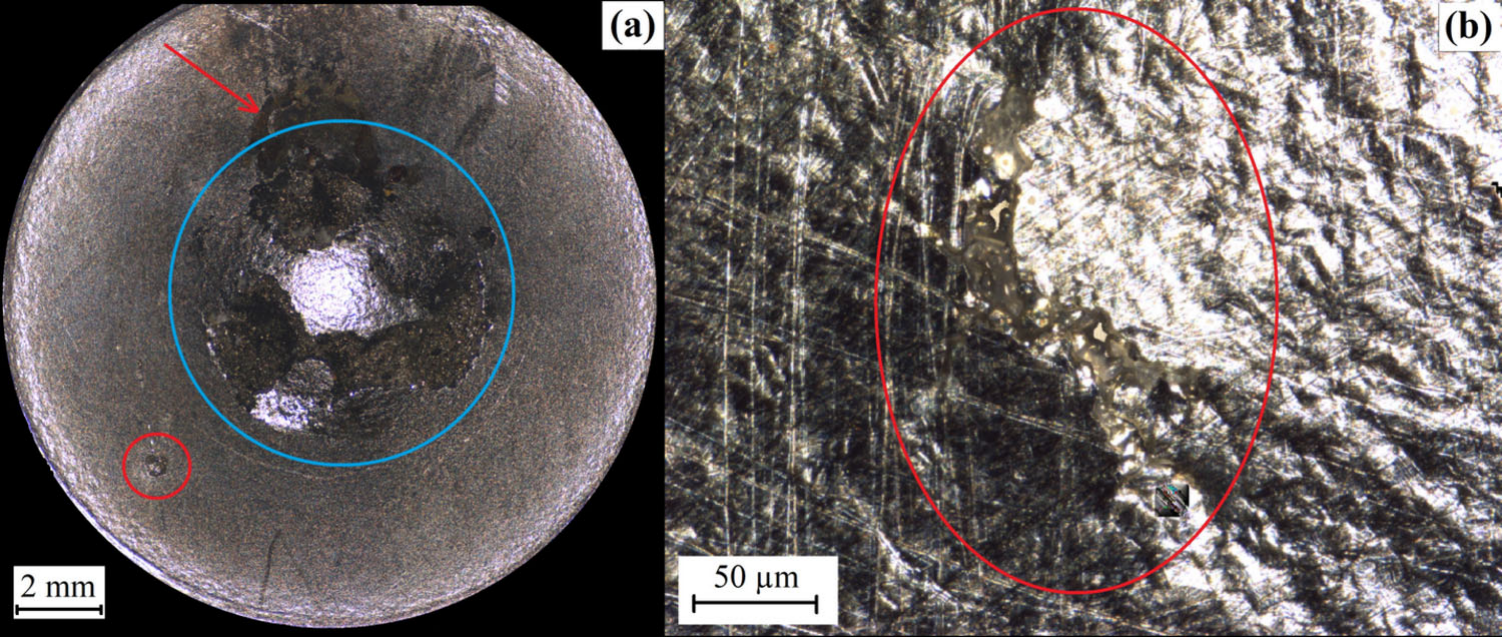
Texture images of (a) the whole surface of sample 1 (with the bore area circled in blue), where the special corroded sites were highlighted in red, and (b) the cracked region (highlighted in the red circle) of sample 3

**Fig. 12:**
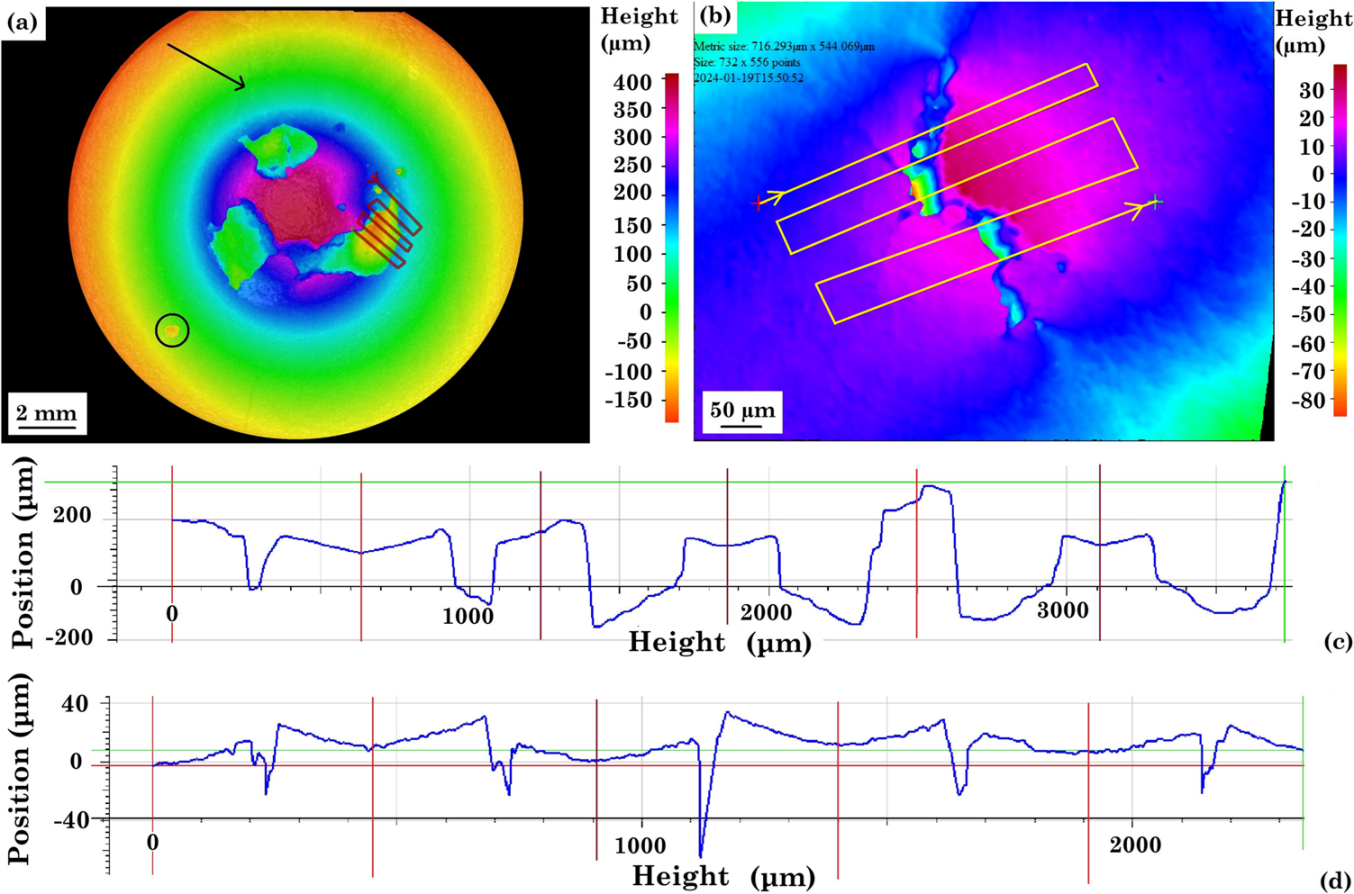
Optical profilometry 3D maps of (a) corroded surface of sample 1 with special corroded region marked in black and a line scan shown in red and (b) cracked region of sample 3 with a line scan shown in red; (c) height profile of the line scan across sample 1 and (d) height profile of the line scan across sample 3

### Optical Profilometry

Figure 11 shows the texture images of sample 1 and sample 3, which were obtained by stitching the images taken at different focal lengths. Figure 11 (a) shows sample 1 mostly suffers from general corrosion, and several pitting corrosion sites can be found on the sample surface. Most corrosion appears in the inner region of the sample that corresponds to the bore region of the sample holder, but there is some corrosion out of the centre region. A pit (highlighted in the red circle) with a diameter of 500 $\upmu$m was initiated between the sample and the holder. An area that is highlighted by the red arrow shows a clear corrosion pattern, which is caused by the flow of the corrosive solution. The strong current flowed into the crevice between the sample and the holder and heavily corroded region with a width of 3 mm, which had the same width as the channel in the specimen holder. It shows it is vital to control the solution temperature of the test to reduce the possibility of other forms of corrosion. The test can also be improved by changing the flow of the corrosive solution to a more dispersed way, so the sample can be exposed to the environment more uniformly. The major stress corrosion cracking site of sample 3 is shown in Fig. 11 (b), where the crack is highlighted in the red circle. Similar to previous optical images, the cracks are very difficult to distinguish from the deformation caused by the load. Apart from the crack, no other forms of corrosion are observed on the sample surface.

Figure 12 shows the optical profilometry 3D maps of the corroded and cracked surface of sample 1 and sample 3, and both the samples have a similar maximum deformation of 500 ± 20$\upmu$m. In sample 1, the region that shows the sign of crevice corrosion (highlighted by the red arrow in Fig. 11 (a)) does not show a significant height difference in the 3D map, which means the corrosion is only superficial. The pitting corrosion location (marked in the black circle) is relatively shallow with a depth of 50 $\upmu$m. Only some cracks near a void (shown in Fig. 6 (c)) are mapped due to the lower resolution, and they are all shallower than the voids with a maximum depth of no greater than 50 $\upmu$m. Figure 12 (c) shows the voids of sample 1 have an average depth of 230 ± 76 $\upmu$m, and the deepest area has a depth of 335 $\upmu$m. The major stress corrosion crack of sample 3 is shown in Fig. 12 (b), and the maximum deformation of the sample is only 40 $\upmu$m. The crack has an average depth of 48 ± 17 $\upmu$m, and the maximum depth of the crack is 92 $\upmu$m. Additionally, the sample surface surrounding the crack is open to release the energy. Some cracks are closed, and the depths of them cannot be measured. As this is an optical surface method, the deeper closed cracks and crack branching cannot be mapped, and other techniques such as X-ray tomography can be used to observe SCC beneath the surface.

## Conclusions

In this study, a novel small punch test setup was built that can accelerate the stress corrosion cracking process of a small thermally sensitised stainless steel disc sample by circulating a corrosive solution. A mechanical preliminary test was performed on a sample to failure in the air, and a yielding load of 210 N was found. In corrosion trial experiments, different corrosive environments were tested and compared to find an ideal solution to accelerate the corrosion process. Due to the complex loading condition, a finite element model was used to analyse the stress distribution of the sample under different loads. When a load of 100 N or 200 N was applied, the maximum tensile stress appeared at the centre of the lower sample surface, and the stress was beyond the yield strength of the material. Four samples were tested in the new SPT setup by circulating a solution with 1000 ppm sodium thiosulphate and 100 ppm hydrochloric acid and characterised by SEM and optical profilometry. The sample suffered from different degrees and forms of corrosion due to the testing temperatures. At higher temperatures, all forms of corrosion including general corrosion and pitting corrosion were initiated in sample 1 and sample 2, where volumes of materials were attacked and forming voids on the sample surface. In sample 3, a lower temperature of 45 $^{\circ }$C only accelerated the initiation of IGSCC and IGC, and further analyses showed that subsurface crack propagation may be different from surface cracking. Finally, sample 4 failed after a long-duration test, and several potential IGSCC sites, which caused the fracture, were identified on the failed sample.

To implement the testing method on irradiated AGR fuel cladding, the samples can be cut from the end cap of the fuel pin by EDM. A custom-made sample holder can be used to prepare the sample by an automated grinding and polishing machine. The rig should be modified for easy operation by a robot arm in the hot cell, and grips can be introduced to assemble the rig. The corrosive solution can be monitored by a sensor and refilled with an automated syringe. Several improvements can be made in future works. Non-uniform corrosion appeared on the sample surface due to the narrow nozzle of the circulation tube, which can be improved by changing the location or the size of the tube connectors. A graph showing the relationship between the SCC initiation time and other factors such as the chemical concentration and temperature, the surface preparation and the loading condition can be developed by testing the samples under different combinations of conditions to standardise the experiment.

Overall, the corrosion small punch test presents a new method for developing stress corrosion cracks from a small sample. With special procedures for irradiated materials introduced, the test can better understand the SCC behaviours of the spent AGR fuel cladding and improve storage safety.
